# Urethra Sparing With Target Motion Mitigation in Dose-Escalated Extreme Hypofractionated Prostate Cancer Radiotherapy: 7-Year Results From a Phase II Study

**DOI:** 10.3389/fonc.2022.863655

**Published:** 2022-03-29

**Authors:** Carlo Greco, Oriol Pares, Nuno Pimentel, Vasco Louro, Beatriz Nunes, Justyna Kociolek, Joep Stroom, Sandra Vieira, Dalila Mateus, Maria Joao Cardoso, Ana Soares, Joao Marques, Elda Freitas, Graça Coelho, Zvi Fuks

**Affiliations:** ^1^ Department of Radiation Oncology, Champalimaud Centre for the Unknown, Lisbon, Portugal; ^2^ Memorial Sloan Kettering Cancer Center, New York, NY, United States

**Keywords:** SABR, SBRT, prostate cancer, dose–response, dose-painting, organ at risk (OAR), endorectal balloon

## Abstract

**Purpose:**

To explore whether the rectal distension-mediated technique, harnessing human physiology to achieve intrafractional prostate motion mitigation, enables urethra sparing by inverse dose painting, thus promoting dose escalation with extreme hypofractionated stereotactic ablative radiotherapy (SABR) in prostate cancer.

**Materials and Methods:**

Between June 2013 and December 2018, 444 patients received 5 × 9 Gy SABR over 5 consecutive days. Rectal distension-mediated SABR was employed *via* insertion of a 150-cm^3^ air-inflated endorectal balloon. A Foley catheter loaded with 3 beacon transponders was used for urethra visualization and online tracking. MRI-based planning using Volumetric Modulated Arc Therapy - Image Guided Radiotherapy (VMAT-IGRT) with inverse dose painting was employed in delivering the planning target volume (PTV) dose and in sculpting exposure of organs at risk (OARs). A 2-mm margin was used for PTV expansion, reduced to 0 mm at the interface with critical OARs. All plans fulfilled D_mean_ ≥45 Gy. Target motion ≥2 mm/5 s motions mandated treatment interruption and target realignment prior to completion of the planned dose delivery.

**Results:**

Patient compliance to the rectal distension-mediated immobilization protocol was excellent, achieving reproducible daily prostate localization at a patient-specific retropubic niche. Online tracking recorded ≤1-mm intrafractional target deviations in 95% of treatment sessions, while target realignment in ≥2-mm deviations enabled treatment completion as scheduled in all cases. The cumulative incidence rates of late grade ≥2 genitourinary (GU) and gastrointestinal (GI) toxicities were 5.3% and 1.1%, respectively. The favorable toxicity profile was corroborated by patient-reported quality of life (QOL) outcomes. Median prostate-specific antigen (PSA) nadir by 5 years was 0.19 ng/ml. The cumulative incidence rate of biochemical failure using the Phoenix definition was 2%, 16.6%, and 27.2% for the combined low/favorable–intermediate, unfavorable intermediate, and high-risk categories, respectively. Patients with a PSA failure underwent a ^68^Ga-labeled prostate-specific membrane antigen (^68^Ga-PSMA) scan showing a 20.2% cumulative incidence of intraprostatic relapses in biopsy International Society of Urological Pathology (ISUP) grade ≥3.

**Conclusion:**

The rectal distension-mediated technique is feasible and well tolerated. Dose escalation to 45 Gy with urethra-sparing results in excellent toxicity profiles and PSA relapse rates similar to those reported by other dose-escalated regimens. The existence of intraprostatic recurrences in patients with high-risk features confirms the notion of a high α/β ratio in these phenotypes resulting in diminished effectiveness with hypofractionated dose escalation.

## Introduction

A deeper understanding of tumor biology has progressively advanced the potentials of tumor cure in primary organ-confined human prostate cancer with radiation therapy. For instance, escalation of conventionally fractionated tumor dose has been shown to render improved local control, mitigating distant metastatic dissemination in a dose-dependent manner (1–3). However, a 15-year update of outcomes in patients treated with dose-escalated 81–86.4 Gy revealed significantly reduced freedom from biochemical prostate-specific antigen (PSA) failure [biochemical recurrence-free survival (bRFS)] (4) compared to previously published 7-year outcomes (2). The delayed manifestation of treatment failures is due to the phenotypic prostate cancer biology, expressed as slowly proliferating and late-responding tumor clonogens associated with a low linear quadratic (LQ) α/β ratio (5, 6).

In 1999, Brenner and Hall (5) suggested that prostate cancer had an α/β ratio of 1.5 Gy (95% CI 0.8–2.2 Gy), confirmed by several large-scale studies establishing ratios within the range 1–2 Gy (7–11). There is, however, emerging evidence that increasing dose per fraction in the hypofractionated mode may be associated with an increase in the *α*/*β* ratio (12, 13). Nonetheless, it is generally accepted that a low *α*/*β* ratio is a basic biological tenet of prostate cancer response to dose fractionation, with therapeutic implications (14).

These findings spurred the exploration of hypofractionated radiation treatment schedules in prostate cancer. Over the past two decades, several large prospective phase III non-inferiority trials compared classical fractionation with iso-Biologically Effective Dose (BED) schedules of moderate (≥20 fractions of 2.4–3.4 Gy) or extreme (4–7 fractions of 5–8 Gy) hypofractionation (14–18), confirming similar ≥5-year bRFS and late grade ≥2 urinary [genitourinary (GU)] and bowel [gastrointestinal (GI)] toxicities between the control and the experimental arms (15–19).

The encouraging outcomes of the non-inferiority trials have promoted a multitude of phase I–II extreme hypofractionation studies with large variations in dose per fraction. A recent meta-analysis of 2,142 patients treated with extreme hypofractionated regimens (33.5–40.0 Gy in 4–5 fractions; 88% receiving 5 fractions) rendered a 7-year bRFS of 87.2% and 82.4% in low- and intermediate-risk patients, respectively (20). Grade ≥3 late GU and GI toxicities were 2.4% and 0.4%, respectively. Similar favorable outcomes were reported by other meta-analyses (21, 22), confirming this therapeutic approach as a standard of care in low- and intermediate-risk patients (23). Recently, a PSA kinetics analysis reported greater prostate ablation and PSA decay with dose escalation up to 40 Gy (5 × 8 Gy) but not beyond allegedly due to the association with distant progression rather than intraprostatic recurrence in the event of PSA relapse at higher doses (24). Additionally, there may be a progressively diminished advantage in increasing dose/fraction as the *α*/*β* ratio may increase as a function of fraction size, resulting in a putative saturation of the dose–response in biochemical control with dose-escalated hypofractionation (13).

However, a recent dose escalation study of 257 patients treated with extreme hypofractionation (five fractions of 6.5, 7.0, 7.5, and 8.0 Gy) included a prostate biopsy assessment at 2 years post-SBRT (25). In 40 patients (15.6%), the biopsies were positive for viable tumor, decreasing in positivity rate in accordance with the four escalating treatment dose levels (37.5%, 21.4%, 19.4%, and 10.9%, respectively). Unfavorable intermediate- or high-risk disease was significantly associated with the occurrence of a positive biopsy. Importantly, only 57% of patients with positive biopsies exhibited evidence of a biochemical relapse within the first 5 years. Furthermore, the study also indicated that extreme hypofractionation with 5 × 8 Gy may be a suboptimal dose in the unfavorable category.

Dose escalation beyond 5 × 8 Gy has been addressed in a multi-institutional phase I/II trial of low- and intermediate-risk disease employing 5 fractions of 9, 9.5, or 10 Gy (26). While the 3-year bRFS was excellent at 98% (26), late GI toxicity was severe, with 6/71 (6.6%) of the patients developing grade 4 late toxicity. Insertion of a peri-rectal polyethylene glycol (PEG) hydrogel spacer systematically reduces the rectal dose and late GI damage in normofractionated prostate cancer patients (27, 28), and it was recently proven effective in a phase II study of 5 × 9 Gy (29). At a median follow-up of 48 months, there were no grade ≥3 GI toxicities, while grade 2 toxicity was initially observed in 14.3% at a median of 11.4 months, completely resolved by year 3 (29). However, the use of a hydrogel spacer does not resolve other concerns associated with prostate cancer radiotherapy, such as the high rates of urethral late grade ≥2 toxicity (30), and the treatment uncertainties associated with an unpredictable mobility of the prostate target during treatment delivery (31).

Here, we review our experience with the use of a novel approach to treat prostate cancer with extreme hypofractionated stereotactic ablative radiotherapy (SABR). We update herein our experience with the use of a unique protocol of rectal distension-mediated prostate immobilization, permitting precise negative dose painting to spare the organs at risk (OARs), with particular emphasis on the intraprostatic urethra. The current update of our initial published observations renders new information on the therapeutic response of different clinical subtypes of human prostate cancer.

## Materials and Methods

### Patients

This is a progress report of an ongoing institutional review board (IRB)-approved non-randomized Phase II study of extreme hypofractionated SABR employing five daily fractions of 9 Gy in patients with organ-confined adenocarcinoma of the prostate (clinicaltrials.gov NCT02761889). All participants signed an informed consent. The present update, consisting of 444 patients (**Table 1**), a 2-fold increase over the previously reported cohort, includes patients treated between June 2013 and December 2018 with a minimum follow-up of 36 months.

**Table 1:** Patient and tumor characteristics.

Characteristics	(n=444)
Age, year	
median (IQR)	70.3 (65.5-74.4)
iPSA, ng/mlL	
median (IQR)	7.1(5.6-10.4)
Gland size, cm 3	
median (IQR)	46.7 (35.1-65.1)
IUSP Grade, n (%)	
Group 1 (3+3)	70 (15.8)
Group 2 (3+4)	234 (52.7)
Group 3 (4+3)	103 (23.2)
Group 4 (4+4)	29 (6.5)
Group 5 (4+5)	8 (1.8)
T-stage, n (%)	
Tlc	28 (6.3)
T2a	106 (23.9)
T2b	124 (27.9)
T2c	182 (41.0)
NCCN Risk, n (%)	
Low	18 (4.1)
Favorable intermediate	103 (23.2)
Unfavorable intermediate	270 (60.8)
High	53 (11.9)
ADT n (%)	162 (36.4)

PSA, Prostate Specific Antigen; iPSA, initial PSA; ADT, androgen deprivation therapy; IQR, interquartile range; mo, months.

### Treatment Planning and Delivery

Patient setup, treatment planning, and treatment delivery were previously described in detail (32, 33). Briefly, patients were planned and treated in a supine position with leg fixation after catheterization with a 12-French gauge (4-mm diameter) Foley catheter with 3 embedded beacon transponders for intrafractional target tracking (Calypso, Varian Medical Systems, Palo Alto, CA, USA). The Foley catheter was also used to guide segmentation of the whole length of the prostatic urethra for dose reduction. Rectal distension-mediated prostate immobilization was achieved by insertion of an endorectal balloon (Rectal Pro, QLRAD Inc., FL, USA) inflated with 150 cm^3^ of air. The insertion of the catheter and endorectal balloon was performed by a dedicated nurse before each session, and the patient was relieved of the endoluminal devices after the completion of the session. To avoid the risk of urinary infection, all patients received prophylactic ciprofloxacin daily during treatment and for 3 days after completion. This technique is based on understanding the physiology of prostate mobility, detailed in the *Discussion* section. A CT and a T2W 3D MR scan were acquired in treatment position.

The fused image sets were used to delineate the target volume and OARs. The planning target volume (PTV) consisted of the clinical target volume (CTV) (the prostate and the proximal two-thirds of the seminal vesicles) with an anisotropic 2-mm expansion margin, reduced to 0 mm at interface with the rectal wall, the bladder, the urethra wall (defined as a 2-mm expansion around the catheter), the urogenital diaphragm (UGD), and the neurovascular bundles (NVBs). Inverse dose painting allowed effective OAR sparing, which was predicated on a reproducible high-precision positioning of the target and all OARs at every treatment session as a result of the organ motion mitigation protocol. The urethral wall was negatively dose painted to fulfill D_1cc_ <36 Gy. The other main OAR constraints were: D_50%_ <22.5 Gy and D_1cm_^3^ <36 Gy for the rectal wall and D_50%_ <22.5 Gy and D_1cm_^3^ <40.5 Gy for the bladder. Priority was given to OAR sparing, but for the PTV, a D_mean_ ≥45 Gy and a near-minimal dose D_98%_ >36 Gy were pursued.

Plans were optimized using penalties to control PTV dose coverage and dose constraints to OARs with the progressive resolution optimizer (PRO v10.0.28-v13.7.14 in Eclipse, Varian Medical Systems, Palo Alto, CA, USA), calculated with the analytical anisotropic algorithm (AAA v10.0.28-v13.7.14). A 10-MV Flattening Filter Free (FFF) beam energy and 4 VMAT arcs were used in all cases. Treatment was delivered on a linear accelerator with a 2.5-mm leaf-width High Definition Multi Leaf Collimator (HDMLC) (TrueBeam STx or EDGE, Varian Medical Systems, Palo Alto, CA, USA). Treatment plans were quality assured before the first treatment session using an ArcCHECK phantom (Sun Nuclear Corp., FL, USA) to confirm that they fulfilled the gamma (3%/3 mm) passing rate >90% objective according to American Association of Physicists in Medicine (AAPM) guidelines.

**Figure 1:**
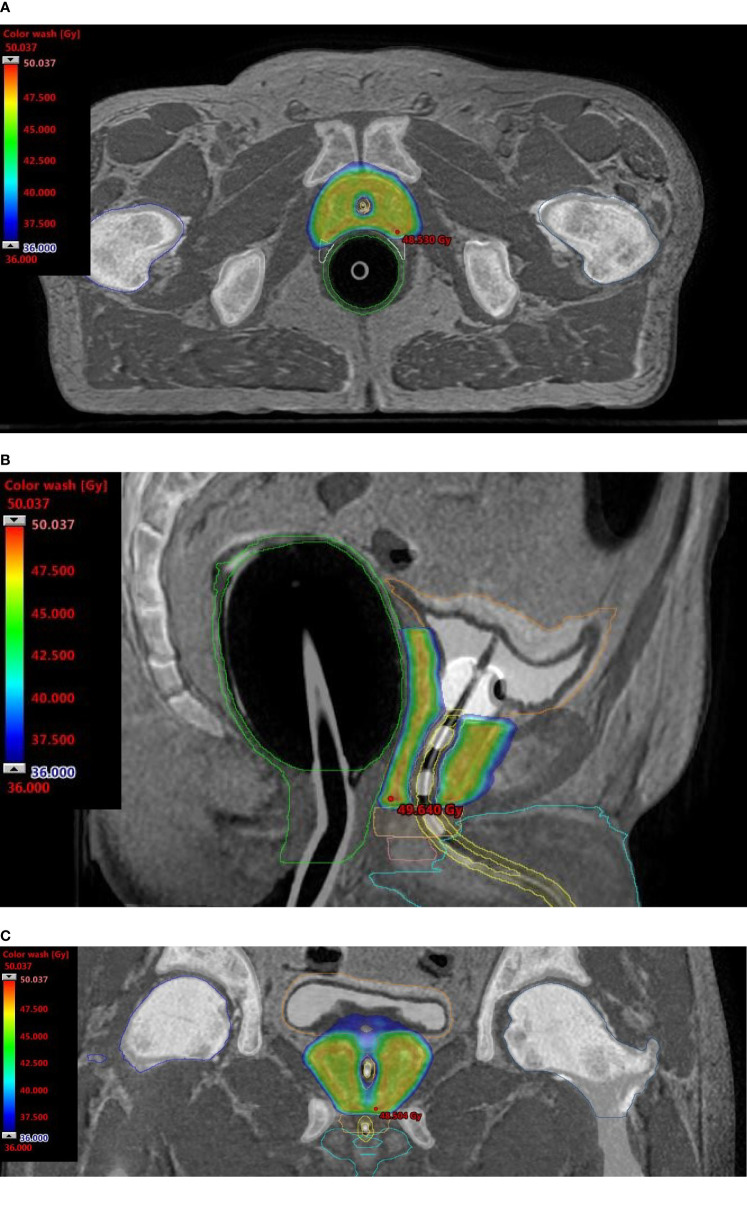
Dosimetric plans of a patient treated with rectal distension-mediated 5 × 9 Gy extreme hypofractionated SABR. Fused CT-MR image sets show dose-sculpted distributions along the urethra, rectal wall, urogenital diaphragm, and neurovascular bundles. Color-wash dose distributions are shown on the axial **(A)**, sagittal **(B)**, and coronal **(C)** planes. An intraurethral Foley catheter loaded with 3 beacon transponders is visible on the longitudinal plane.

Onboard cone-beam computed tomography (CBCT) matching ensured reproducible patient setup and target localization. If discrepancies of ≥1 mm in translation or ≥1 degree in rotation were detected, corrections were applied *via* a 6-degrees of freedom couch (PerfectPitch 6-DoF Couch, Varian Medical Systems, Palo Alto, CA, USA). When beacon transponder signals exceeded an accepted 2-mm deviation threshold for ≥5 s, treatment was interrupted, and treatment target position was redefined by repeat CBCT. Patients received treatment daily over 5 consecutive days. **Figure 1** shows dose distributions representative of the typical plan.

### Characterization of Biochemically Relapsing Patients

Patients with a biochemical relapse were assessed with a ^68^Ga-labeled prostate-specific membrane antigen (PSMA)-11 PET/CT scan to determine the existence of intraprostatic vs. extraprostatic progression. An activity of 2 MBq/kg of patient body weight of ^68^Ga-PSMA-11 was administered using an automatic injector (INTEGO™, MEDRAD), and images were acquired at 45–60 min post-injection. The PET/CT (Gemini TF, Philips) scan was acquired with a low-dose CT (120–140 kV, 60 mA per rotation) from the skull base to the upper third of the thighs. PET data were obtained thereafter with a sequence of 6–8 bed positions, always on 3D mode for 1.5–3 min on average per bed position. In addition to visual analysis, quantitative SUV evaluation was performed within the volumetric region of interest (Extended Brilliance Workspace algorithm NM 2.0 AB-V5.4.3.40140, Philips). The Standardized Uptake Value (SUV) for the voxel with the highest activity concentration (SUV_max_) was recorded. Institutional criteria for quantitative assessment ^68^Ga-PSMA uptake were: SUV_max_ of lesion/SUV_max_ of normal prostate or surrounding tissues >4.0 was considered positive; 2.0–4.0, suspicious; and <2.0, negative.

In addition to ^68^Ga-PSMA-11 PET/CT, multiparametric MRI scans of the prostate and biopsy were employed where appropriate.

### Toxicity and Quality of Life Assessment

Toxicity [National Cancer Institute Common Terminology Criteria for Adverse Events (NCI CTCAE) v.4.0] was assessed posttreatment at 1 month and every 3 months to 12 months (± 4 weeks), at every 6 months for years 2–5, and annually thereafter. Acute toxicity was defined as any adverse event occurring within 90 days from the beginning of treatment. International Prostate Symptom Score (IPSS), Expanded Prostate Cancer Index Composite-26 (EPIC-26), and International Index of Erectile Function Questionnaire (IIEF) questionnaires were completed at baseline and at the same time points posttreatment as above.

### Statistical Methods

The primary endpoints of the study were incidence of treatment-related acute and late adverse events and PSA outcomes. Actuarial bRFS, GU and GI toxicities, and patient-reported quality of life (QOL) scores were computed from the end of treatment using the Kaplan–Meier method. For each EPIC domain, a summary score was calculated at each of the study time points. Wilcoxon signed-rank test analysis was used to assess differences in QOL scores compared to baseline, and the significance of the mean changes over time was assessed by paired mixed-effects analysis. The clinically meaningful decline in QOL [minimally important difference (MID)] was defined as one-half of the standard deviation from baseline for each domain. Univariate analysis of relevant variables was performed using the Cox proportional hazards regression method. Hazard ratio (HR) and 95% confidence intervals (CIs) were obtained, and the level of statistical significance was set at alpha = 0.05. Statistical computations were performed using the GraphPad *Prism 7.0* software (Prism Inc., Reston, VA, USA).

## Results

The characteristics of the 444 patients are summarized in **Table 1**. Median follow-up time was 58 months [interquartile range (IQR), 44.3–78.8]. Twelve patients succumbed to comorbidities without evidence of disease at a median follow-up time of 39.8 months, and another 29 were lost to follow-up at a median time of 34.6 months. Patients were stratified according to NCCN criteria. At the discretion of the referring physician, 162 patients received androgen deprivation therapy (ADT) for a median duration of 6 months (IQR, 3–6).

All patients were strictly planned and treated with the 150-cm^3^ air-filled balloon, in full compliance with the rectal distension-mediated prostate immobilization, and the beacon transponder-loaded Foley catheter technique. Plan objectives and dosimetric results are summarized in **Table 2**. Due to the high inherent dose heterogeneity of the plan dose prescriptions, PTV doses are reported in accordance with the International Commission on Radiation Units and Measurements (ICRU) recommendations (34) as D_50%._ All plans fulfilled a D_50%_ ≥45.0 Gy and a D_95%_ ≥40.5 Gy. Patient adherence to the protocol was excellent, and all completed the planned 5 sessions over 5 consecutive days (i.e., Monday through Friday).

**Table 2:** Plan objectives and dosimetric results.

Plan dosimetry	Plan objective	Median	mean	IQR
PTV				
D_50%_ (Gy)	≥45.0	46.6	46.6	46.4-46.7
D_mean_ (Gy)	≥45.0	45.8	45.8	45.6-46.0
D_95%_ (Gy)	≥40.5	40.6	40.4	40.0-41.2
D_2%_ (Gy)	≤48.2	47.9	47.9	47.8-48.1
D_98%_ (Gy)	≥38.2	38.4	38.6	38.1-39.1
V_45Gy_ (%)	≥80	80.6	80.8	77.7-84.0
V_40.5Gy_ (%)	≥95	94.6	95.1	93.9-96.2
Urethral wall				
D_2%_ (Gy)	≤40.5	38.7	38.8	38.4-39.2
D_1cm 3_ (Gy)	≤36.0	33.7	34.4	33.8-34.9
Bladder				
D_2%_ (Gy)	≤40.5	36.8	37.5	28.2-40.6
D_5o%_ (Gy)	≤22.5	14.5	10.5	8.0-12.4
D_1cm3_ (Gy)	≤40.5	38.6	38.9	38.2-39.5
Rectal wall				
D_2%_ (Gy)	≤42.8	35.5	35.2	35.1-35.8
D_5%_ (Gy)	≤40.5	32.7	33.3	32.2-33.8
D_5o%_ (Gy)	≤22.5	9.9	14.4	7.27-17.6
D_1cm3_ (Gy)	≤36.0	35.6	35.3	35.0-35.5
UGD				
D_2%_ (Gy)	≤42.8	35.9	37.4	33.3-39.7
Penile bulb				
D_2%_ (Gy)	≤36.0	3.3	2.4	1.8-3.5
D_1cm_ (Gy)	≤22.5	2.0	1.6	1.3-2.2
NVBs				
D_2%_ (Gy)	≤45.0	39.6	41.4	39.0-44.6
D_5o%_ (Gy)	≤31.5	30.1	31.3	28.5-33.8
Femoral heads				
D_2%_ (Gy)	≤22.5	12.9	12.8	5.5-20.9

PTV, Planning Target Volume; D_mean_. mean dose; D_2%_, D_5%_, D_5o%_D_95%_, D_98%_, minimum dose to n% of the structure; V_458y_, V_40.58y_, percentage of structure receiving 45Gy or 40.5Gy (100% and 90% of the prescription dose); D1cm^3^, dose to 1 cm^3^ of the structure; UGD, urogenital diaphragm; NVB, neurovascular bundles.

### Prostate-Specific Antigen Outcomes

**Figure 2:**
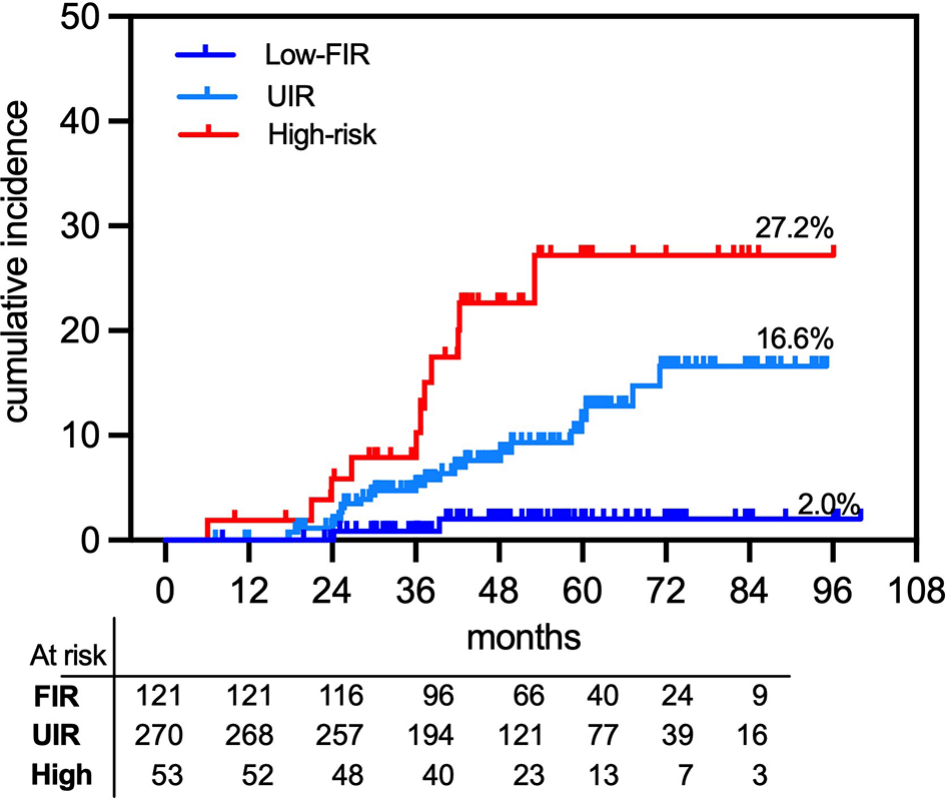
Actuarial prostate-specific antigen (PSA) relapse in 444 organ-confined primary prostate cancer treated with rectal distension-mediated 5 × 9 Gy SABR. Patient groups are defined as combined low-risk and favorable intermediate-risk (FIR), unfavorable intermediate-risk (UIR), and high-risk (High) patients.

**Figure 3:**
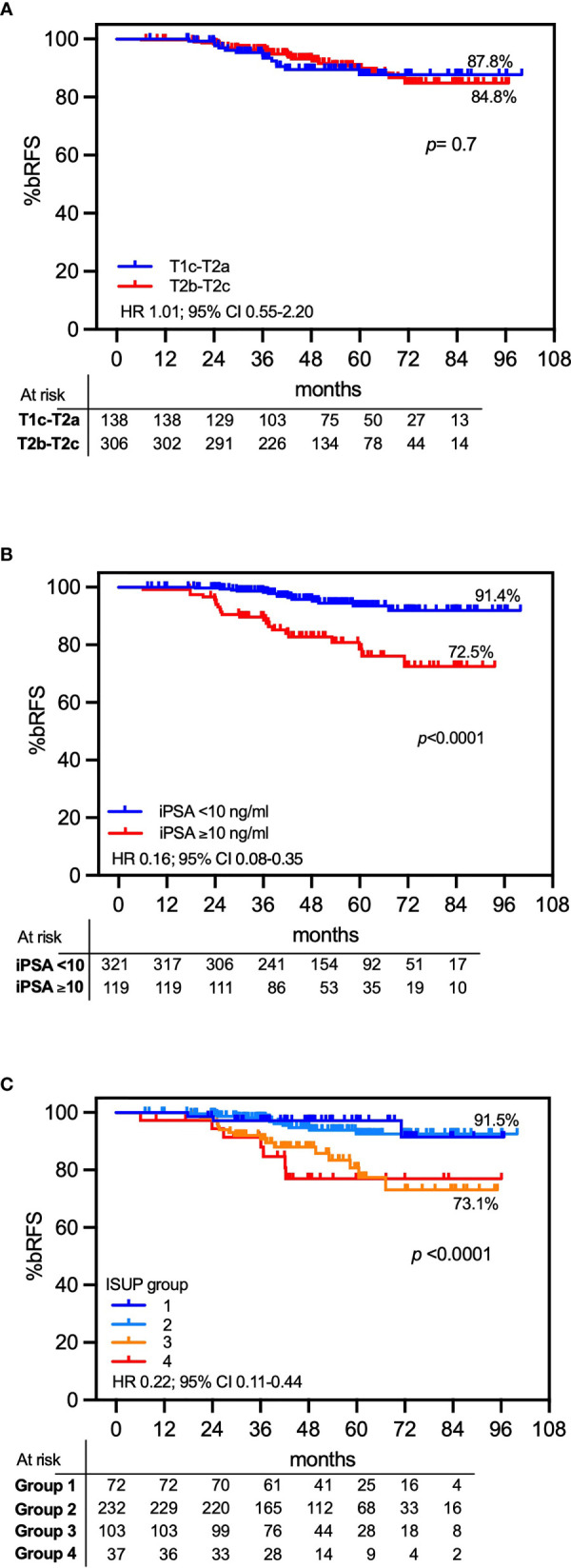
Seven-year actuarial PSA relapse-free survival (bRFS). Actuarial bRFS is presented as a function of T stage **(A)**, initial PSA **(B)**, and biopsy ISUP grade group **(C)**.

A total of 37 patients developed a Phoenix-defined (nadir +2 ng/ml) PSA relapse at a median time of 36.1 months (IQR, 25.2–42.3). The 7-year cumulative incidence rate of PSA failure was 13.8% for the entire cohort. **Figure 2** shows that the cumulative incidence rates of PSA failures were 2% vs. 16.6% for the combined low and favorable intermediate-risk (FIR) groups vs. the Unfavorable Intermediate-Risk (UIR) group (*p* < 0.005; HR 0.32, 95% CI 0.14–0.71) and 27.2% for the high-risk group (unfavorable intermediate-risk vs. high-risk, *p* = 0.01, HR 0.31, 95% CI 0.12–0.77). **Figure 3** shows associations of pretreatment characteristics with bRFS probability. MRI-defined T-stage was not correlated with bRFS (87.8% vs. 84.8% for T1c-T2a vs. T2b-T2c, respectively; *p* = 0.7; HR 1.01; 95% CI, 0.55–2.20; **Figure 3A**), while pretreatment PSA (iPSA) was significantly associated with bRFS (91.4% vs. 72.5% for iPSA <10 vs. ≥10 ng/ml, respectively; *p* < 0.0001; HR 0.16; 95% CI, 0.08–0.35; **Figure 3B**). Biopsy ISUP grade group 1 vs. 2 did not significantly differ in bRFS probabilities nor did ISUP group 3 vs. 4. However, the combination of Groups 1 and 2 vs. 3 and 4 resulted in significantly different bRFS probabilities (91.5% vs. 73.5%, *p* < 0.0001; HR 0.22; 95% CI, 0.11–0.44; **Figure 3C**).

### Androgen Deprivation Therapy in UIR and High-Risk Patients

**Figure 4:**
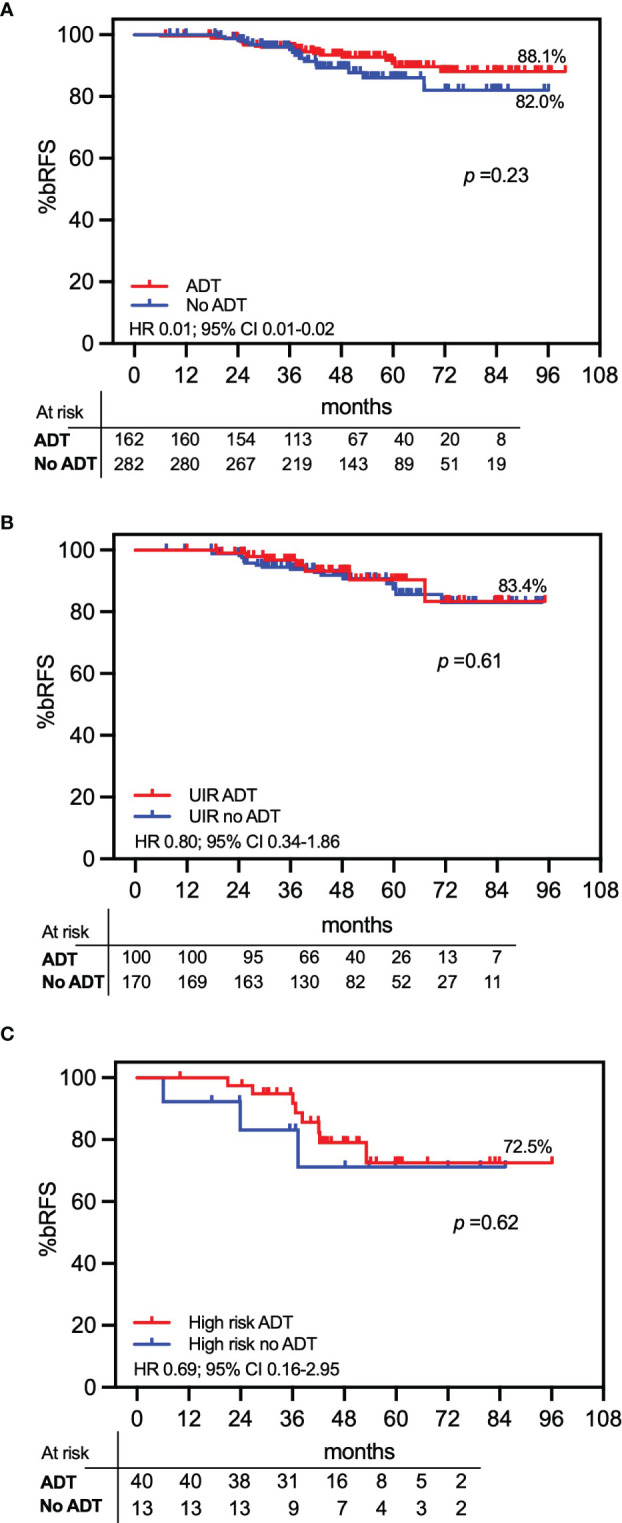
Actuarial PSA relapse-free survival (bRFS) by androgen deprivation therapy (ADT). Patients treated with ADT are compared with patients not receiving ADT. **(A)** The total study population of 444 patients; **(B)** unfavorable intermediate-risk (UIR) patients; **(C)** high-risk patients.

The use of ADT was not one of the primary study objectives, and patients were not randomized for ADT administration, which was employed at the discretion of the referring physician. Overall, the 7-year bRFS probability for patients who received ADT was 88.1% vs. 82.0% for those who did not (*p* = 0.023; HR 0.01; 95% CI, 0.01–0.02; **Figure 4A**). Additionally, subset analysis of UIR and high-risk patients who received ADT vs. those who did not showed no statistically significant differences between the two UIR subgroups (83.3% vs. 83.4%, respectively; *p* = 0.61; HR 0.80; 95% CI, 0.34–1.86; **Figure 4B**) and high-risk subgroups (72.5% vs. 72.3%, respectively; *p* = 0.62; HR 0.69; 95% CI, 0.16–2.95; **Figure 4C**).

### Prostate-Specific Antigen Kinetics in No Androgen Deprivation Therapy Patients

**Figure 5:**
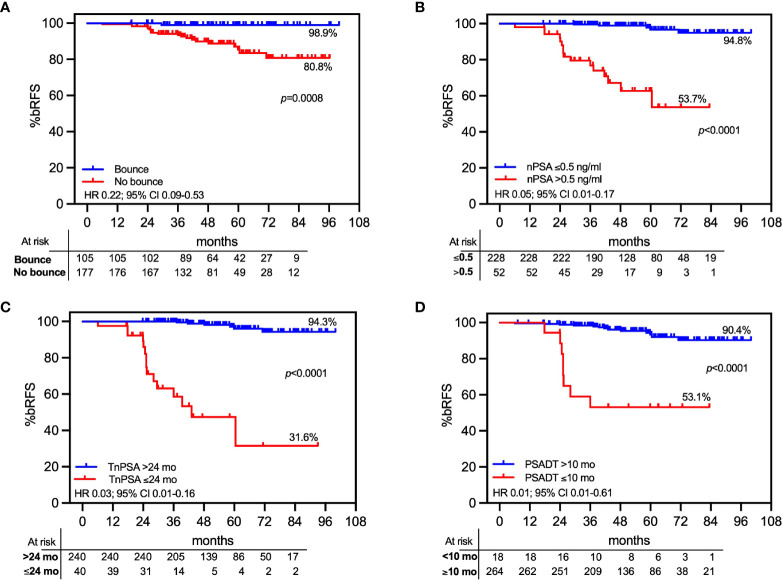
Actuarial PSA relapse-free survival (bRFS) by landmarks of PSA relapse predictors. Only patients who did not receive androgen deprivation therapy (ADT) were included in the analyses. Patients classified by **(A)** benign PSA bounce; **(B)** nadir PSA cutoff at 0.5 mg/ml; **(C)** time to nadir PSA (TnPSA) with a cutoff time point at 24 months; **(D)** PSA doubling time with 10 months as cutoff point.

Whereas the present study adopted tight PTV safety margins and a urethra-sparing approach, we explored established landmarks of PSA relapse predictors to ensure that the treatment protocol did not negatively affect outcomes. In the 282 patients who were not exposed to ADT, PSA gradually decreased to a median nadir of 0.19 ng/ml (IQR, 0.09–0.37), and the 3-year median PSA was 0.30 ng/ml (IQR, 0.20–0.32). Benign PSA bounces (>0.2 ng/ml over previous nadir) were observed in 36.5% (103/282) of cases and had a median magnitude of 0.57 ng/ml (IQR, 0.32–097). The median time to bounce was 12 months (IQR, 8.9–17.5), and the median duration was 3 months (IQR, 3–9). PSA bounces were significantly correlated with bRFS in this cohort (98.9% vs. 80.8% for patients with vs. without a bounce; *p* = 0.0008; HR 0.22; 95% CI, 0.09–0.53; **Figure 5A**). A PSA nadir (nPSA) <0.5 ng/ml was significantly correlated with an improved probability of bRFS (94.8% for nPSA <0.5 ng/ml vs. 53.7% for ≥0.5 ng/ml; *p* < 0.0001; HR 0.05; 95% CI, 0.01–0.17; **Figure 5B**). Time to nPSA (TnPSA) was significantly associated with decreased bRFS using 24 months as a cutoff point (94.3% vs. 31.6% for TnPSA <24 vs. ≥24 months, respectively; HR 0.03; 95% CI, 0.01–0.16; **Figure 5C**). Likewise, a 24-month PSA doubling time (PSADT) ≥10 months was associated with significantly decreased PSA relapse rates (90.4% vs. 53.1% for PSADT ≥10 vs. <10 months, respectively; HR 0.01; 95% CI 0.01–0.61; **Figure 5D**).

### ^68^Ga-PSMA PET/CT Characterization of Prostate-Specific Antigen Relapses

**Figure 6:**
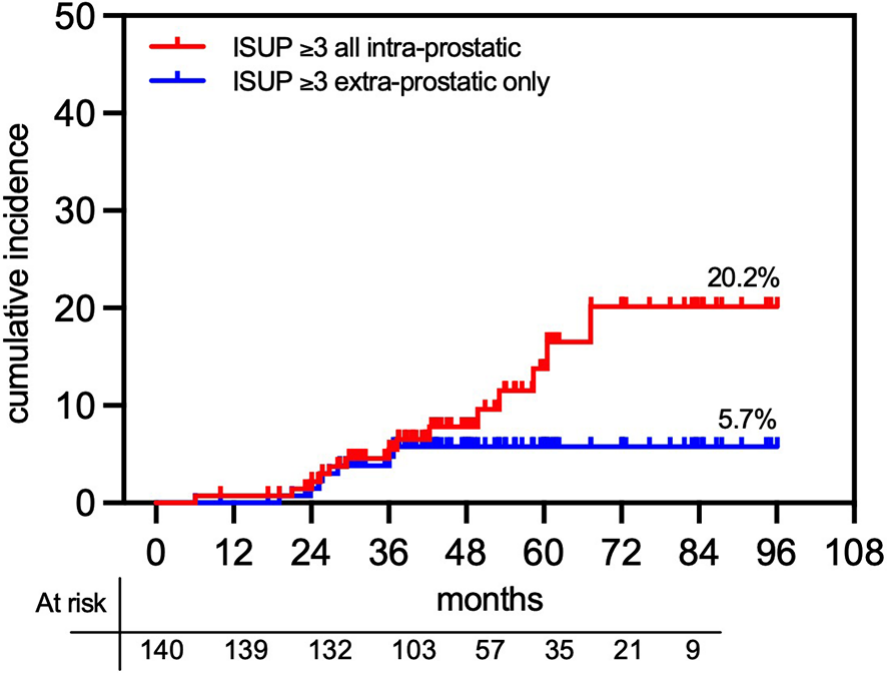
Cumulative actuarial incidence of post-SABR prostate cancer relapse in histological ISUP ≤3 group. Relapse was detected by ^68^PSMA PET/CT scanning detecting intraprostatic (± extraprostatic) lesions vs. extraprostatic only tumor lesions.

**Figure 7:**
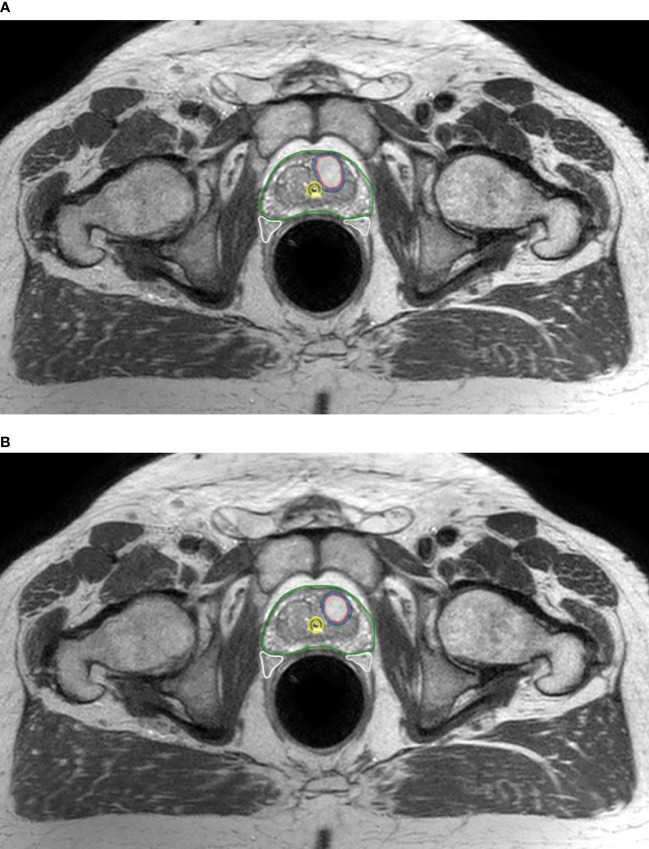
^68^PSMA-detected relapse in a patient who received 5-fraction SABR. **(A)** Fused ^68^PSMA-PET/CT and planning MRI show pretreatment location of ^68^PSMA-detected dominant intraprostatic lesion (DIL) encompassed within the CTV and receiving the full 45 Gy prescription dose; **(B)** fused ^68^PSMA-PET/CT acquired at the occurrence of biochemical relapse with the planning MRI scan shows persistence/recurrence of the DIL at the initial site.

To determine whether PSA relapse in ISUP groups 3 and 4 was associated with extraprostatic spread, we employed ^68^Ga-PSMA PET/CT at the time of PSA failure. Scans were performed in 35 of the 37 patients exhibiting a PSA relapse. Median PSA at the time of relapse was 3.70 (IQR 2.39–5.20). In one patient, the ^68^Ga-PSMA scan was inconclusive. **Figure 6** shows that for ISUP groups ≥3, the actuarial 7-year cumulative incidence rate of all ^68^Ga-PSMA-detected intraprostatic recurrences [Local relapse (LR)] was 20.2% vs. 5.7% extraprostatic only progression. Of the 34 patients with positive ^68^Ga-PSMA scans, 73.5% (25/34; 2 FIR, 15 UIR, and 8 high-risk) had evidence of persistent tracer uptake at the site of the pretreatment dominant lesion, 4 of whom (1 UIR and 3 high-risk) also exhibited nodal involvement. In contrast, 26.5% (9/34) of patients had evidence of extraprostatic dissemination only. The overall 7-year actuarial cumulative incidence rate of developing a ^68^Ga-PSMA-detected intraprostatic or extraprostatic relapse was 9.9% vs. 4.6%, respectively. **Figure 7** shows an instance of a ^68^Ga-PSMA-detected intraprostatic relapse at the same site of the initial dominant lesion for an ISUP grade 3 tumor. Biopsy ISUP grade ≥3 (i.e., Gleason primary pattern 4) was significantly associated with the likelihood of a ^68^Ga-PSMA-detected intraprostatic relapse (20.2% vs. 5.6% for ISUP groups ≥3 vs. ≤2, respectively; *p* = 0.004; HR 0.28; 95% CI, 0.12–0.68; not shown), also differing in extraprostatic dissemination only (8.6% vs. 2.6% for ISUP groups ≥3 vs. ≤2, respectively; *p* = 0.0003; HR 0.11; 95% CI, 0.03–0.37; not shown).

### Adverse Events

**Table 3** summarizes the incidence of acute and late adverse events. Grade 1 acute urinary (GU) symptoms peaked at 1 month posttreatment with an overall incidence of 19.8% (n = 88) largely consisting of dysuria and frequency. Acute grade 2 GU toxicity was observed in 6.8% (n = 27), including 4 cases (0.9%) of retention that needed catheterization during the first week posttherapy. There were no cases of grade 3 GU toxicity. There was no statistically significant association between baseline IPSS score ≥15 and the likelihood of developing acute grade 2 GU toxicity (Fisher’s exact test *p* = 0.4). Acute grade 1 rectal (GI) toxicity occurred in 6.5% (n = 29) of cases and was largely represented by tenesmus. The incidence rate of acute grade 2 GI was 0.5% (n = 2), and there were no instances of acute grade 3 GI events.

**Table 3:** Acute and late toxicities.

% (n)	Acute			Late		
	G1	G2	G3	G1	G2	G3
**Any GU**	19.8% (88)	6.8% (27)	0% (0)	13.1% (58)	4.5% (20)	0.2% (1)
Dysuria	11.9% (53)	4.5% (20)		5.4% (24)	1.6% (7)	
Frequency/urgency	8.1% (36)	1.1% (5)		5.2% (23)	1.3% (6)	
Nocturia	2.7% (12)			1.3% (6)	0.9% (4)	
Retention	1.1% (5)	1.8% (8)		0.7% (3)	0.2% (1)	
Incontinence	1.1% (5)	0.4% (2)		0.4% (2)	0.2% (1)	
Hematuria	0.4% (2)	0.4% (2)		3.2% (14)	0.4% (2)	0.2% (1)
**Any GI**	6.5% (29)	0.5% (2)	0% (0)	3.2% (14)	1.1% (5)	0% (0)
Tenesmus	3.8% (17)	0.2% (1)		0.7% (3)		
Rectal bleeding	2.2% (9)	0.2% (1)		2.0% (9)	1.1% (5)	
Diarrhea	0.9% (4)	0.2% (1)		0.7% (3)		

**Figure 8:**
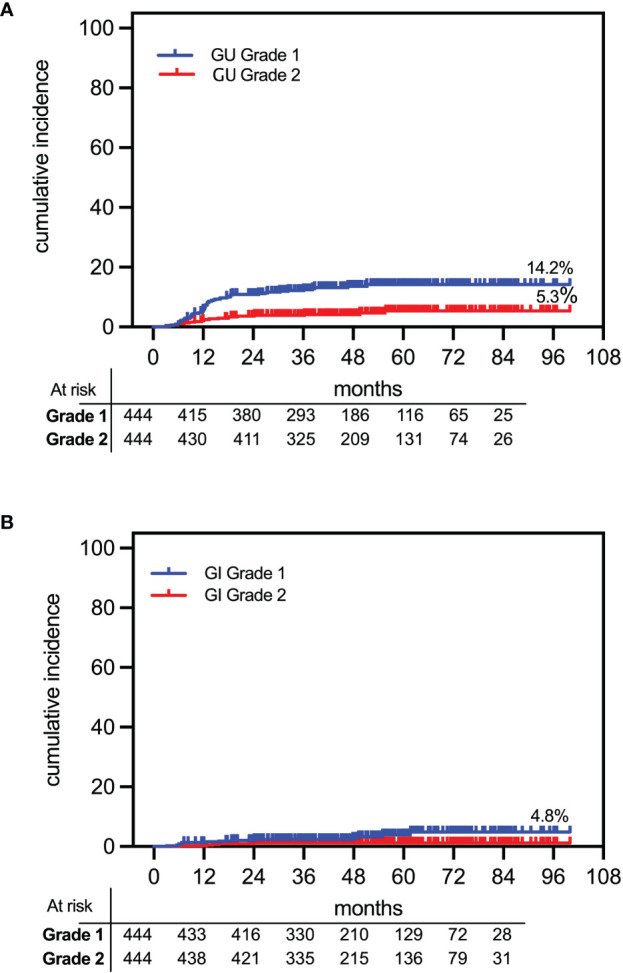
Cumulative actuarial incidence of OAR toxicities following rectal distension-mediated 5 × 9 Gy SABR. The 7-year late grade 1 and 2 toxicities are shown for **(A)** urinary (GU) and **(B)** bowel (GI) toxicities.

Late grade 1 and grade 2 GU toxicities occurred, respectively, in 13.1% (n = 58) and 4.5% (n = 20) of patients. There was only one instance of grade 3 toxicity (0.2%) presenting at 4.3 months posttherapy as severe hematuria requiring transfusion. Median time to late GU toxicity was 12.4 months (IQR, 9.1–17.3). The actuarial cumulative incidence rates of late GU adverse events were 14.2% and 5.3%, respectively, for grade 1 and grade ≥2 (**Figure 8A**). None of the patients in this cohort developed late urinary retention requiring catheterization.

Late GI toxicity occurred at a median of 14.1 months (IQR, 6.2–41.9) posttherapy, consisting of 3.2% (n = 14) grade 1 and 1.1% (n = 5) grade 2 rectal bleeding events. There were no instances of grade 3 rectal toxicity. The actuarial cumulative incidence rates of late GI adverse events were 4.8% and 1.1%, respectively, for grade 1 and grade 2 (**Figure 8B**).

### Patient-Reported Quality of Life

**Figure 9:**
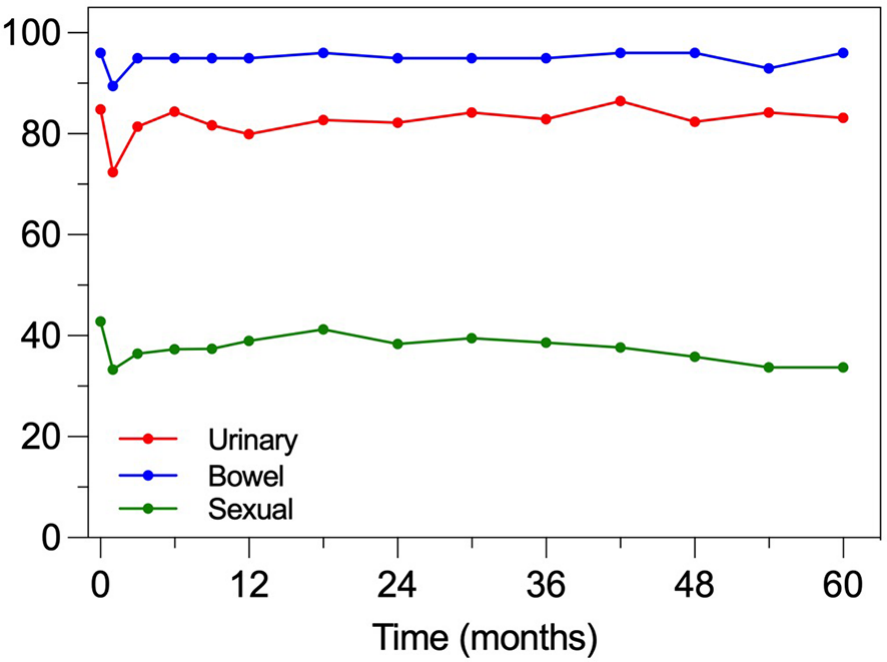
Median EPIC-26 summary scores for the urinary, bowel, and sexual domains. EPIC-26 summary scores range between 0 and 100, with higher scores indicating better QOL.

Patient-reported QOL measures showed a transient decline in all three EPIC-26 summary score domains at 1 month after treatment, recovering at 3 months (**Figure 9**). The clinically meaningful decline in QOL was defined as one-half the standard deviation of each of the domain baseline summary scores (MID). Median changes from baseline and proportions of patients with declines above the MID for the three EPIC-26 domains at each of the study time points are summarized in **Table 4**. As far as the urinary domain is concerned, the overall magnitude of the declines over time was relatively small and the proportions of patients with urinary domain declines >MID were relatively constant over time, except for a second transient increase (34.7% of patients) at 12 months posttreatment, representing the occurrence of a temporary self-limiting pelvic flare phenomenon. Notwithstanding, in the present series, the RTOG 0938 urinary domain meaningful endpoint for the tolerability and safety of prostate SBRT (defined as declines from baseline to 1 year >2 points in ≤60% of patients) was fulfilled at 47.0%, confirming the favorable toxicity profile of the present approach also when using such a stringent endpoint metric.

**Table 4:** EPIC-26 summary score changes and proportions of patients with declines above the minimally important difference.

EPIC-26	1 mo	3mo	6mo	9mo	12 mo	18 mo	24mo	30 mo	36 mo	42mo	48mo	54mo	60 mo
**Urinary domain** median =88 SD =12.4													
Median change (IOR)	-8 (-21, 0)	0 (-6, 4)	0 (-6, 4)	0 (-9, 4)	-2 (-8, 4)	0 (-9, 4)	-2 (-7, 4)	0 (-7, 6)	0 (-8, 6)	0 (-6, 6)	0 (-7, 4)	0 (-7, 6)	-2 (-9, 4)
MID 6.2 points													
Proportion with decline >MID	54.40%	24.70%	23.10%	29.10%	34.70%	29.10%	26.40%	25.90%	26.30%	24.00%	26.00%	31.00%	33.30%
**Bowel domain** median =96 SD =8.5													
Median change (IQR)	-3 (-11, 0)	0 (-5, 3)	0 (-5, 2)	0 (-5, 2)	0 (-6, 2)	0 (-5, 3)	0 (-5, 3)	0 (-7, 3)	0 (-5, 3)	0 (-5, 2)	0 (-3, 3)	0 (-5, 2)	0 (-6, 2)
MID 4.2 points													
Proportion with decline >MID	43.60%	27.30%	28.60%	29.00%	31.00%	28.20%	30.40%	26.50%	30.20%	25.00%	26.00%	25.00%	23.60%
**Sexual domain** median =42 SD =26.9													
Median change (IQR)	-6(-19, -3)	-2(-15, 3)	-4(-16, 3)	0(-13, 6)	-2(-14, 6)	-2(-12, 8)	-2(-13, 8)	-2(-13, 6)	-4(-13, 8)	-7(-18, 5)	-6(-19, 6)	-6(-17, 3)	10(-23, -2)
MID 13.4 points													
Proportion with decline >MID	36.50%	29.40%	27.90%	24.20%	25.10%	22.50%	24.90%	23.10%	29.10%	34.70%	35.40%	34.60%	37.70%

The bowel domain had minimal changes over time. The RTOG 0938 bowel domain meaningful endpoint for tolerability and safety (defined as declines from baseline to 1 year >5 points in ≤55% of patients) was also met (25.9% in the present series), underlining the effectiveness of the present technique in bowel QOL preservation.

The sexual domain had the largest absolute changes between baseline and the study time points. However, the summary scores were only marginally reduced compared to baseline until month 36 posttreatment, after which the magnitude of the decline and the proportions of patients with changes above MID gradually increased, suggesting that sparing of the NVBs may contribute to the sexual domain QOL preservation.

## Discussion

The present study provides compelling evidence for the efficacy of the rectal distension-mediated prostate cancer SABR. The ability to reproducibly immobilize the prostate within the same patient-specific anatomical niche, at the same 3D configuration, promotes the basic tenet of ablative radiotherapy, namely, high-precision tumor targeting with OAR preservation. Although post-SABR prostate biopsies were not performed in this study, an early use of ^68^Ga-PSMA PET/CT provided an approach to detect LRs within the irradiated prostate. The low-risk/FIR patients exhibited an actuarial 7-year incidence of biochemical failure of 2%, with 2 patients failing with LR. In contrast, LRs occurred with an incidence of approximately 20% in the UIR and high-risk patients. Whereas there were no discrepancies in treatment planning or delivery in these patients, this observation confirms the existence of human prostate cancer subphenotypes that exhibit resistance to the 5 × 9 Gy schedule, consistent with recent reports of an α/β ratio range of 1.3–11.1 Gy, derived from analysis of known LQ variables in subgroups of hypofractionated prostate cancer patients (12).

### The Physiology of Prostate Organ Motion

The introduction of the rectal distension-mediated technique in prostate cancer SABR was derived from an understanding of prostate physiology as a mobile organ. The anatomical location of the human prostate at a resting state is in the inferior–posterior section of the pelvic diaphragm (35). It has been generally believed that the strategic location at the pelvic outlet exposes the gland to random dislocation by rectal gas or urinary bladder filling (36). In fact, cine-MRI studies showed that high-volume gas passing through the rectum induces a prostatic gland translocation of up to 12 mm, subsequently returning to its steady-state location (37), and online tracking technology disclosed unpredictable 3–10-mm prostate organ displacements during radiation treatment delivery in approximately 20% of treatment sessions (38–40), engendering uncertainties in prostate tumor targeting (41, 42).

However, anatomical studies have indicated that the human prostate cannot independently drift, as it is restricted by complex anatomical interactions with adjacent pelvic organs. At its base, the prostate is attached to the bladder neck, while at the apex, the *levator ani puborectalis* muscle tightly engulfs the gland at the level of the anorectal ring (43). Posteriorly, the prostate body and seminal vesicles blend through Denonvilliers’ fascia to the *ampulla recti* (44, 45), an actively mobile structure (46, 47). Hence, prostate mobility largely represents a bystander phenomenon to the physiology of the rectum.

Stretching of the rectal wall activates efferent neuronal sensorimotor signals that coordinate the *levator ani puborectalis* muscle function in regulating anorectal junction patency (46, 47). The *levator ani puborectalis* originates at the posterior surface of the pubic ramus and runs along the right and left of the prostate/rectum complex, forming a sling around the posterior rectal wall just proximal to the anorectal junction. The muscle is permanently contracted under baseline conditions (postural reflex), forming a rectal angulation that obliterates passage of intrarectal contents (46, 47). When stretching of the rectal wall occurs, efferent neurosignals relax the *levator ani puborectalis* postural reflex (47), unfolding the loop of the rectum *via* its expansion along a superior–anterior axis, concomitantly relocating the linked prostate along the same vector.

This functional paradigm suggests an approach to immobilize the prostate for a certain period of time by introducing an endorectal air-inflated balloon during each SABR session, harnessing the physiology of the rectal/prostate mobility. A body of literature shows that 40–100 cm^3^ of air-inflated endorectal balloon reduces prostate intrafractional motion, but some intrafractional motion still occurs (48–50). The suboptimal outcome of ≤100 cm^3^ air-filled balloons raises the question of whether stretch receptor signals may be insufficient. In fact, human data indicate that rectal sensorimotor stretch receptors adapt with time to an isobaric rectal wall stretch, returning to baseline function at a rate that is inversely related to the isobaric volume distending the rectum (51–53). Hence, we posit that immobilization of the prostate during radiation treatment delivery requires a sustained state of near-maximal resolution of the *puborectalis* postural reflex and of the anorectal angulation, avoiding the risk of rectal stretch adaptation. Consistent with this notion, studies of escalated air volume inflation of intrarectal balloons reported that at the low air-volume range, patients consistently report mild, if any, sensation of rectal distension, while at volumes exceeding approximately 150 cm^3^, patients reported an intolerable urge to evacuate (54, 55). We posited that the transition volume from tolerable to urgency/intolerable sensation might define an adequate state of near-maximal resolution of the postural reflex/anorectal angulation, which might optimize prostate immobilization during SABR treatment delivery.

### Rectal Distension-Mediated Prostate Immobilization

We have tested this hypothesis in the first 189 patients of the present phase II clinical study of 5 × 9 Gy SABR (32, 41). An initial balloon-volume tolerance study was performed in the first 15 patients during simulation, demonstrating that in our air-filled endorectal balloon system, the highest tolerated air filling was 150 cm^3^ (32). The rectal distension-mediated treatment protocol was employed using this volume (41), and full transponder/Linac logs from 886 treatment sessions were systematically analyzed (41). Accurate alignment of the anatomy between the planning image scan and the CBCT at the time of delivery is of paramount importance. Urethra sparing is achieved if the curvature of the intraprostatic urethra is perfectly matching, often requiring minor manual adjustments of the catheter and endorectal balloon. Of course, any small readjustment must be confirmed by a new CBCT before final registration is approved by the treating physician. Therefore, mean preparation time from online tracking inception to reference CBCT acquisition was 14.1 ± 11 min, and an average of 3.7 ± 1.7 CBCTs were required for final reference registration (41).

The overall mean session time was 19.5 ± 12 min, including 5.4 ± 5.9 min for actual treatment delivery after reference CBCT acquisition, registration, and approval (41). Treatment interruptions due to deviations requiring a realignment CBCT occurred in 6% of sessions, prolonging session time to a mean of 14.5 ± 8.4 min.

Posttreatment analysis of the log data showed that the majority of >2-mm intrafraction motions occurred in the superior-inferio (SI) (7.6%), anterior-posterior (AP) (2.8%), and left-right (LF) (3.2%) directions, indicating a relative stability along these axes (41). All detected deviations were managed either by temporary treatment interruptions until they resolved spontaneously or by target realignment following new CBCT acquisitions. Temporary deviations were rare during the first 10 min (1.4%), gradually increasing to 3.8% by 15 min, minimally prolonging the overall treatment delivery time (41). The rectal distension-mediated approach rendered recapitulation of the daily repositioning of the prostate/OAR complex into an anatomically confirmed same patient-specific retropubic niche, within a maximum standard deviation of 1.5 mm (32, 41), enabling accurate delivery of the high-heterogeneity treatment plans.

### Toxicity Profile

The rectal distension-mediated prostate immobilization approach used here was well tolerated by all patients and resulted in a low cumulative incidence of acute and late grade ≥2 urinary and rectal toxicities. These favorable outcomes are to be attributed to the meticulous efforts deployed during MRI acquisition, treatment planning, and treatment delivery in ensuring maximal anatomical reproducibility. Hence, the strict implementation of stringent dose constraints for the OARs *via* negative dose painting and the tight PTV expansion margin as used in this study have rendered the low OAR toxicity rates reported herein. Additionally, the online tracking with 2-mm threshold guaranteed the applicability of such tight margins by way of correction for intrafraction motion.

While the overall 7-year cumulative incidence rate of urinary late grade ≥2 toxicity in the present study was 5.3%, studies that did not employ urethra sparing reported a significantly higher dose-dependent urinary toxicity. Helou et al. (56) reported that Radiation Therapy Oncology Group grade ≥2 late urinary toxicity sharply increases to 48% in patients receiving 40 Gy. Zhang et al. (57) showed that V42 Gy >2 cc was associated with significantly increased grade ≥2 urinary toxicity. Zelefsky et al. (58) showed that the risk of RTOG ≥2 urinary toxicity increases in a stepwise fashion in a dose escalation study (23.3%, 25.7%, 27.8%, and 31.4% for the dose levels 32.5, 35, 37.5, and 40 Gy, respectively). A recent dosimetry modeling of the risk of urinary toxicity based on the maximum urethral dose metric (MUDM; calculated in EQD_2_) has shown that each increase of 1 Gy corresponds to a 1% increase in risk of grade ≥2 and 0.2% in grade 3 late urinary toxicity (30). While our experience is consistent with this model, the strict constraints employed in our study (maximal dose to the urethral wall D_2%_ ≤40.5 Gy and D1cm^3^ ≤36 Gy) resulted in 4.7% and 0.2% CTCAE grade ≤2 and grade 3 urinary toxicities, respectively. Similar results have been reported by studies adopting urethral constraints of 34–35 Gy (late grade ≥2: 3.8%–8.3%) (59–61). Thus, our data provide compelling evidence that dose escalation in a 5-fraction SABR regimen can be safely pursued provided stringent urethra sparing and accurate target anatomical localization are deployed.

The GI toxicity profile in this study compares favorably with recently reported toxicity outcomes of dose-escalated extreme hypofractionation. For instance, Musunuru et al. (62) reported a >20% vs. 8% grade ≥2 CTCAE GI toxicity in patients treated with 40 vs. 35 Gy, respectively. In a dose escalation trial, 10% of patients treated with 50 Gy experienced grade 3–4 rectal toxicity (26). Dosimetric analysis showed a strong association between V39 Gy >35% of rectum circumference and the risk of late bowel toxicity. In addition to the anatomical reproducibility and accuracy of the technique deployed here, we believe that the maximally tolerated stretching of the rectum by the air-filled endorectal balloon reduces the exposure of most of the mucosa of the rectal wall, permitting the fulfilment of the strict D1cm^3^ ≤36 Gy constraint. Thus, our results compare favorably with recent reports employing hydrogel spacers with dose-escalated regimens similar to ours (29, 58). Therefore, we maintain that the use of the hydrogel spacer, apart from being invasively inserted, only affords protection on the rectal mucosa and does not prevent or mitigate prostate organ motion, thereby foregoing the opportunity of accurate urethra and NVB sparing. The physician-reported toxicity profiles are corroborated by the favorable long-term patient-reported QOL outcomes. Nonetheless, the reported QOL changes following ultrahigh-dose hypofractionation reflect an existence of low-grade chronic symptoms that may be of particular interest due to the lack of severe adverse events observed with SBRT in several series (63). These observations highlight the importance of QOL evaluations in prostate cancer therapy.

### Impact of Dose Escalation on Local Control

The dose prescription of the present study translates into a spectrum of high tumor ablative BED when tumors consist of LQ α/β ≤2 Gy functioning clonogens, driving the effectiveness of extreme hypofractionation. Consistent with this notion, only 2/121 (1.6%) of low-risk/FIR patients exhibited a PSA relapse, both associated with ^68^Ga-PSMA*-*detected LRs, while the 7-year bRFS rate was stable at 98%. In contrast, however, the same treatment regimen employed in the UIR/high-risk category rendered a significantly higher 7-year cumulative incidence of ^68^Ga-PSMA-detected intraprostatic relapses, as well as extraprostatic dissemination. This observation raises questions relative to the relevance of the dogmatic acceptance of a single low-range α/β phenotype in defining the LQ fractionation sensitivity in prostate cancer. In fact, Vogelius and Bentzen (13), while confirming the validity of a functional *α*/*β* ratio of 1.6 Gy, also highlighted an association between increasing dose in hypofractionation schemes and an increase of α/β values, suggesting an existence of *α*/*β* heterogeneity in prostate cancer. Datta et al. (12) confirmed the α/β heterogeneity in hypofractionation studies, ranging between 1.3 and 11.1 Gy. Our ^68^Ga-PSMA PET/CT studies in UIR/high-risk tumors are consistent with this notion, suggesting that the continuous genomic and metabolic drivers of clonal expansion, which confer high-risk clinical features, such as ISUP grade ≥3 phenotypes, might hypothetically confer clones of high α/β in fractionation responses. Such biologic phenotypes would render hypofractionation BEDs that are significantly lower than the ablative BED ≥_2_. Attempts to reach an ablative iso-BED_2_ in 5-fraction whole-prostate SABR would require an unattainable increase in the fractional dose due to a high risk of OAR toxicity. Recent evidence, however, is emerging on the feasibility, safety, and efficacy of a simultaneous integrated boost (SIB) *via* ^68^Ga-PSMA*-*directed dose painting of dominant intraprostatic lesions (DILs) (64–67). The SIB/DIL approach has been shown to be feasible in prostate cancer treated with extreme hypofractionation (68, 69), but the safety and effectiveness of SIB/DIL as described here will need to be tested in carefully designed clinical trials such as the ongoing Hypofocal-SBRT study (70).

## Conclusion

The present clinical trial provides compelling evidence that the rectal distension-mediated technique affords a non-invasive and safe approach to employ an ablative 5-fraction SABR regimen to treat prostate cancer, albeit maximally effective in low LQ α/β phenotypes. Approximately 20% of UIR/high-risk patients appear to develop locally relapsing, radioresistant high α/β phenotypes. There is, thus, an urgent need for new tools to discern patients who are refractory to dose-escalated 5-fraction SABR and to introduce hypofractionated-based treatment techniques to improve tumor control in this biological setting. Whether the ^68^Ga-PSMA*-*directed SIB/DIL technique might comprehensively ablate clones at high LR risk as part of a 5-fraction whole-prostate SABR remains to be tested.

## Data Availability Statement

The datasets will not be made available due to patient privacy concerns. Requests to access the datasets should be directed to: carlo.greco@fundacaochampalimaud.pt.

## Ethics Statement

The studies involving human participants were reviewed and approved by the Champalimaud Ethics Committee. The patients/participants provided their written informed consent to participate in this study.

## Author Contributions

CG was the lead author who initially developed the concept of the study, participated in data collection, data analysis, article drafting, table/figure creation, and article revision. OP, NP, VL, BN, and JK participated in clinical data collection and data analysis. JS, SV, MC DM, AS, JM, EF, and GC participated in data collection and analysis. ZF is a senior author who participated in data analysis, article drafting, review, and revision. All authors contributed to the article and approved the submitted version.

## Conflict of Interest

The authors declare that the research was conducted in the absence of any commercial or financial relationships that could be construed as a potential conflict of interest.

## Publisher’s Note

All claims expressed in this article are solely those of the authors and do not necessarily represent those of their affiliated organizations, or those of the publisher, the editors and the reviewers. Any product that may be evaluated in this article, or claim that may be made by its manufacturer, is not guaranteed or endorsed by the publisher.
